# Correlative Microscopy of Lamellar Hole-Associated Epiretinal Proliferation

**DOI:** 10.1155/2015/450212

**Published:** 2015-09-03

**Authors:** Denise Compera, Enrico Entchev, Christos Haritoglou, Wolfgang J. Mayer, Felix Hagenau, Jean Ziada, Anselm Kampik, Ricarda G. Schumann

**Affiliations:** Department of Ophthalmology, Ludwig-Maximilians-University, Mathildenstraße 8, 80336 Munich, Germany

## Abstract

*Purpose*. To describe morphology of lamellar hole-associated epiretinal proliferation (LHEP) removed from eyes with lamellar macular holes (LMH). *Methods*. Based on optical coherence tomography data, 10 specimens of LHEP were removed from 10 eyes with LMH during standard vitrectomy. Specimens were prepared for correlative light and electron microscopy (CLEM) using an immunonanogold particle of 1.4 nm diameter that was combined with a fluorescein moiety, both having been attached to a single antibody fragment. As primary antibodies, we used antiglial fibrillary acidic protein (GFAP), anti-CD45, anti-CD64, anti-*α*-smooth muscle actin (*α*-SMA), and anticollagen type I and type II. *Results*. In LHEP, GFAP-positive cells possess ultrastructural characteristics of fibroblasts and hyalocytes. They represent the major cell types and were densely packed in cell agglomerations on vitreous collagen strands. Epiretinal cells of LHEP rarely demonstrated contractive properties as *α*-SMA-positive myofibroblasts were an infrequent finding. *Conclusion*. CLEM indicates that epiretinal cells in LHEP might originate from the vitreous and that remodelling processes of vitreous collagen may play an important role in pathogenesis of eyes with LMH.

## 1. Introduction

Recently, the term of lamellar hole-associated epiretinal proliferation (LHEP) was introduced by Pang and colleagues to characterize a thick homogenous layer of unusual material on the epiretinal surface in eyes with lamellar macular holes (LMH) [1, 2]. High-resolution optical coherence tomography (OCT) studies have demonstrated that eyes with LMH frequently show this epiretinal proliferation presenting as a highly reflective line with moderately reflective material filling the space between the inner border of the epiretinal proliferation and the retinal nerve fibre layer [3–7].

However, pathogenesis, morphology, and clinical course of eyes with LHEP are poorly understood. By recent OCT studies, the presence of LHEP was shown to be related to the presence of photoreceptor layer defects and poor visual acuity compared to eyes with LMH without LHEP [1, 2, 8]. On OCT examination, LHEP does not appear to have contractive properties [1, 2, 5, 8]. In general, traction forces by conventional ERM become visible as retinal folds that are usually not seen in retinal layers covered by LHEP.

Since there is little detail on cell and collagen composition of LHEP, the aim of this study was to describe morphologic characteristics of LHEP in eyes with LMH by correlative microscopy. Correlative light and electron microscopy (CLEM) was recently proposed using immunonanogold particles of 1.4 nm diameter combined with a fluorescein moiety that both are attached to a single antibody fragment [9–11]. In this study, we used CLEM for improved visualization of cells and extracellular matrix of LHEP [12].

## 2. Materials and Methods

### 2.1. Patient Samples

Surgically excised specimens of LHEP were consecutively harvested from 10 eyes of 10 patients with lamellar macular holes during vitrectomy. All patients required surgery due to the severity of clinical symptoms such as progressive visual loss. Patients' records were reviewed for age, gender, previous ocular surgery, and preoperative history such as trauma. All specimens were obtained from patients who were part of a recent retrospective OCT study reporting on clinical course of 112 operated and nonoperated eyes with lamellar macular holes and macular pseudoholes [8]. Clinical data of patients and volume B-scans of high-resolution OCT examinations (Spectralis OCT, Heidelberg Engineering, Heidelberg, Germany) were reevaluated in order to exclusively include eyes with presence of LHEP. This study was approved by the Institutional Review Board and Ethics Committee and was conducted according to the tenets of the Declaration of Helsinki. Informed consent was obtained from all patients before surgery.

### 2.2. Surgical Procedure

Patients underwent a standard 23-gauge vitrectomy. Surgery was recommended if BCVA decreased to LogMAR 0.3 or more, if BCVA decreased 2 Snellen lines or more during the preoperative follow-up period, and if the patient experienced a significant impairment of the quality of life. If necessary, a posterior vitreous detachment (PVD) was induced by suction with the vitrectomy probe around the optic nerve head. The posterior hyaloid was detached from the retina and PVD was extended to the periphery with the vitreous being removed at least up to the equator. For removal of LHEP, intraocular end-gripping forceps were used. For ILM peeling, a 0.25 mg/mL solution of Brilliant Blue (Brilliant Peel, Fluoron GmbH, Neu-Ulm, Germany) was used. The vitreous cavity was then filled with a tamponade of either 15% hexafluoroethane (C2F6) gas-air mixture, or air, or balanced salt solution. Infrequently, patients were instructed to keep a face-down position for at least 2 days.

### 2.3. Specimen Preparation

For fixation, specimens were placed into 2% paraformaldehyde solution. Indirect immunocytochemistry was performed for all specimens after flat-mount preparation following interference and phase contrast microscopy. If necessary, large specimens were divided into pieces in accordance with their size in order to label excised tissue of each patient with all antibodies. Specimens were incubated with 0.1% pepsin and normal donkey serum. Primary antibodies for glial and retinal cells (antiglial fibrillary acidic protein (anti-GFAP), DAKO, Hamburg, Germany); for hyalocytes (anti-CD45 and anti-CD64, Santa Cruz Biotechnology, Heidelberg, Germany); for myofibroblasts (anti-*α*-smooth muscle actin (anti-*α*-SMA), Santa Cruz Biotechnology, Heidelberg, Germany); and for extracellular matrix (anticollagen type I (anti-col-I), Santa Cruz Biotechnology, Heidelberg, Germany; anticollagen type II (anti-col-II), Biotrend, Cologne, Germany) were added and incubated over night at room temperature. As second antibody, FluoroNanogold (Fab′-fragments, Nanoprobes, Yaphank, NY, USA) was incubated for two hours at room temperature. Postfixation with 2% glutaraldehyde was following.

Preparing negative controls, specimens with large area and multilayered cell proliferation were cut into half in order to use one part for labelling procedures and the other part for negative control preparation. The primary antibody was substituted with both diluent and isotype controls (IgG2a monoclonal mouse antibodies, X0934, DAKO, Hamburg, Germany; M5409, Sigma-Aldrich, Taufkirchen, Germany). All other procedures were identical to the procedures illustrated above.

Flat-mount preparation of LHEP specimens was performed as recently reported [12]. Following fluorescence microscopic analysis (Leica DM 2500, Wetzlar, Germany) at magnifications between ×50 and ×400, specimens were processed for transmission electron microscopy. Specimens were incubated with gold enhancement solution. Postfixation in osmium tetroxide 2% and uranyl acetate as well as dehydration and embedding in Epon 812 was following. Ultrathin sections of 60 nm were obtained by series-sectioning and were contrasted with uranyl acetate and lead citrate. Analysis and imaging of 5 grids (each with 6–9 ultrathin sections) per specimen were performed using a transmission electron microscope Zeiss EM 9 S-2 (Zeiss, Jena, Germany).

## 3. Results

### 3.1. Clinical Data Analysis

This is a series of 10 surgically excised specimens of LHEP obtained during vitrectomy from eyes with lamellar macular holes. Patients' mean age was 70 ± 6 years (median, 70 years; range, 63–82 years). We included four women and six men.

Preoperatively median BCVA of eyes with LMH was LogMAR 0.40 (mean 0.41 ± 0.13 SD) and increased postoperatively to median BCVA of LogMAR 0.30 (mean 0.34 ± 0.19 SD) during a mean follow-up period of 8.6 months (median 10 months; range, 3–15 months). The difference was not statistically significant (Wilcoxon test, *p* > 0.05). In detail, 6 of 10 patients improved visual acuity, whereas 3 of 10 patients lost vision, and in one patient BCVA remained unchanged. From the eyes with LHEP only, 4 of 5 eyes improved BCVA, whereas one of 5 patients lost vision acuity. Considering eyes with a combination of both LHEP and an extrafoveal conventional ERM, 2 of 5 patients improved vision acuity. Two of 5 patients lost BCVA and one patient was stable.

At time of surgery, 8 of 10 eyes were phakic and 2 eyes were pseudophakic. All of the phakic eyes underwent combined vitrectomy with cataract extraction and intraocular lens implantation. Regarding the presence of posterior vitreous detachment (PVD), a complete PVD was seen in 4 of 10 eyes as intraoperatively assessed by the surgeon. A partial PVD was documented in 4 of 10 eyes, and an attached posterior vitreous was found in 2 of 10 eyes. Postoperatively, none of the eyes developed a full-thickness macular hole and no persistent macular edema was noted.

**Figure 1:**
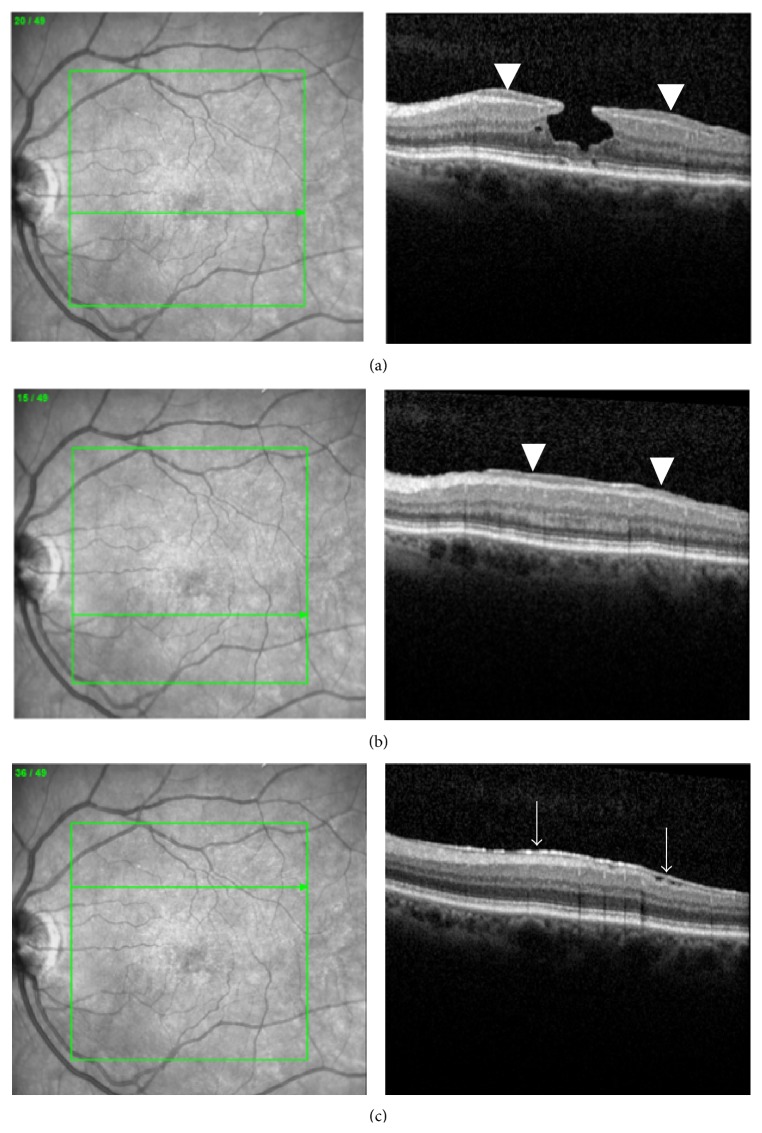
Spectral-domain optical coherence tomography images of a 73-year-old female with lamellar macular hole and lamellar hole-associated epiretinal proliferation (arrowheads) seen (a) at the macular defect and (b) in the parafoveal area. (c) A conventional epiretinal membrane (arrows) was found extrafoveal with some distance to the foveal defect.

In SD-OCT examinations, LHEP was directly located at the macular defect (Figures 1(a) and 1(b)). In half of all eyes, a combination of both LHEP and a conventional ERM with contractive properties was seen. If present, conventional ERM was found extrafoveal with some distance to the foveal defect (Figure 1(c)). Preoperatively, defects of the ellipsoid zone were detected in 8 of 10 eyes (Table 1). In 2 eyes, defects of the external limiting membrane (ELM) were documented. At last follow-up, defects of the ellipsoid zone were seen in 7 of 10 eyes. Discontinuity of the ELM was seen in one eye.

### 3.2. Correlative Light and Electron Microscopy

**Figure 2:**
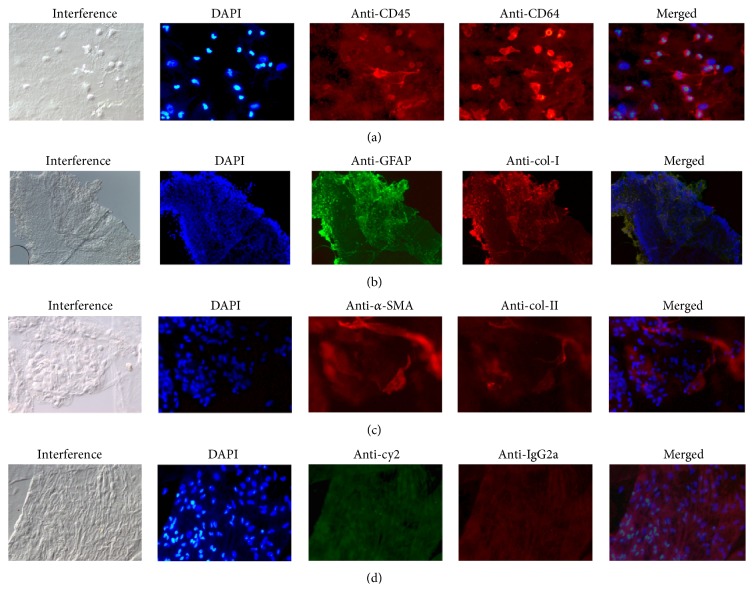
Interference microscopy, cell nuclei staining with 4′,6-diamidino-2-phenylindole, DAPI (blue), and immunocytochemical staining of lamellar hole-associated epiretinal proliferation removed from eyes with lamellar macular holes (LMH). (a) Epiretinal cells show positive immunolabelling with anti-CD45 (red) and anti-CD64 (red) in specimen removed from eyes with LMH. (b) Immunostaining of epiretinal cells seen as a thick homogenous layer positively labelled with anti-GFAP (green) and anticollagen type I (anti-col-I) (red). (c) Immunolabelling with anti-*α*-smooth muscle actin (*α*-SMA) (red) and anticollagen type II (anti-col-II) (red). (d) Negative control specimen with positive cell nuclei staining but no specific immunoreactivity of cell proliferation. (Original magnification: (a) ×400; (b) ×100; (c-d) ×400).

Analysing flat-mounted specimens, positive immunostaining for anti-GFAP and for the hyalocyte cell markers anti-CD45 and anti-CD64 was seen in all eyes with LHEP (Table 1, Figure 2). Anticollagen type I was often positive as well as immunolabelling for anticollagen type II. Moreover, a colocalisation of anti-GFAP with anti-CD64 as well as anticollagen type I was seen in several specimens. In negative control specimens, no specific positive immunostaining was observed.

By transmission electron microscopy, the ILM was characterized by its undulated retinal side and the smooth vitreal side. The ILM was noted in 8 of 10 specimens removed from eyes with LMH. The ILM was clearly differentiated from thick collagen strands.

**Figure 3:**
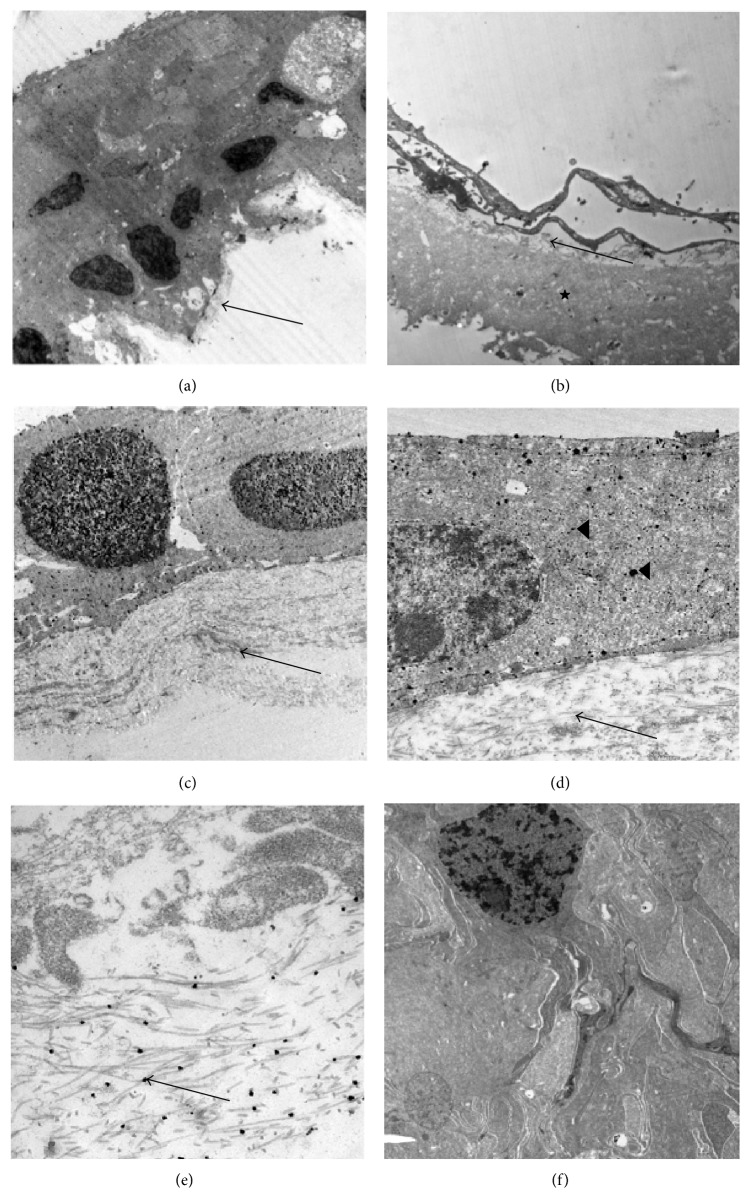
Transmission electron micrographs of lamellar hole-associated epiretinal proliferation (LHEP) with immunonanogold application following gold enhancement preparation procedures. (a) Densely packed cell agglomeration of fibroblasts and hyalocytes situated on a thin strand of vitreous collagen (arrow). (b) Internal limiting membrane (big star) with small vitreous collagen deposits (arrow) and fine cellular processes on the vitreal side. (c, d) Native vitreous collagen (arrow) with GFAP-positive fibroblasts as demonstrated by immunonanogold staining (arrowhead). (e) Small black dots (arrow) represent immunonanogold particles staining collagen type II of vitreous cortex collagen. (f) Negative control specimen with typical dense epiretinal cell proliferation seen as cell agglomeration of fibroblast-like cells. (Original magnification: (a) ×3,000; (b) ×4,400; (c, f) ×7,500; (d) ×18,000; (e) ×55,000).

In epiretinal cell proliferation, fibroblasts and hyalocytes were the predominant cell types (Figure 3). Fibroblasts were characterized by their abundant rough endoplasmatic reticulum, prominent golgi complex, and a fusiform shape of the cell body and nucleus. Hyalocytes were distinguished by their lobulated cell nuclei, intracellular vacuoles, vesicles, and mitochondria as well as long cell fibers. Myofibroblasts containing cell fibers with contractile forces were rarely found. In the collagen matrix, native vitreous collagen (NVC) was predominant and identified as major type of collagen. It is characterized by a regular arrangement of fibrils with a collagen fibril diameter of less than 16 nm. Newly formed collagen (NFC) with irregular fibril arrangement and fibril diameter of more than 16 nm was seen as well. In NVC, fibrous long spacing collagen (FLSC) was frequently found.

Negative controls did show neither specific labelling of cellular structures nor extracellular components by immunonanogold labelling.

## 4. Conclusions

This is the first correlative light and electron microscopic study presenting histopathologic data of LHEP by using application of immunonanogold. Correlative light and electron microscopy was recently reported to improve visualization of cells and extracellular matrix in epiretinal membranes by using FluoroNanogold as secondary antibody. It composes an immunonanogold particle of 1.4 nm diameter that is combined with a fluorescein moiety and a single antibody Fab-fragment [9–12]. By application of immunonanogold particles, we were able to analyse the same cellular and extracellular components of LHEP by fluorescence and electron microscopy.

Lamellar hole-associated epiretinal proliferation was recently suggested to be primarily driven by a proliferation of Müller cells onto the inner retina originating from the middle layers of the retina [1, 2]. In this study, we demonstrated GFAP-positive cells in LHEP. This finding is in accordance with immunohistological results of Parolini and colleagues, who also presented cells with positive immunostaining of anti-GFAP [5]. In the majority of studies, immunoreactivity for GFAP in epiretinal membranes was usually interpreted as an indicator for the presence of glial cells [13]. However, electron microscopy revealed fibroblasts as predominant cell type in this analysis. Hyalocytes were seen as well.

More recent studies demonstrated positive GFAP staining in other cell populations than glia. Of note, fibroblasts and hyalocytes were occasionally described to be GFAP-positive [14–18]. Several species were found to present GFAP-positive hyalocytes, including porcine, pectineal, and bovine hyalocyte cell lines. Therefore, we hypothesize that vitreous derived cells rather than cells of glial origin may play a major role in pathogenesis of LMH with LHEP. In this series, fibroblasts were often seen densely packed in cell agglomerations, mostly situated on vitreous collagen strands. These cell agglomerations did not show signs of contraction. In contrast, myofibroblast-like cells with contractive properties were a rare finding. Our observations are in accordance with SD-OCT examinations demonstrating a thick homogenous layer of unusual material on the epiretinal surface in eyes with lamellar macular holes that does not show contraction signs [1, 2].

Predominance of vitreous collagen in specimens of LHEP was reported by Parolini et al. and has been confirmed by this study [5]. Native vitreous collagen fibrils were arranged as thick collagen strands often dispersed with fibrous long spacing collagen that is known to represent a remodelling process of vitreous collagen [18, 19]. Thus, LHEP appears to primarily consist of vitreous derived cells proliferating on vitreous collagen strands that are marked by degradation and remodelling of collagen components.

In this study, the majority of eyes with LHEP showed defects of the ellipsoid zone in preoperative SD-OCT examinations, which is in accordance with previous reports on disruptions of the outer photoreceptor layer in LMH [2, 8]. Furthermore, recent studies demonstrated that the presence of LHEP correlated with defects of the ellipsoid zone and the ELM layer [8, 20, 21]. However, it is still unknown why eyes with LMH do not respond to ERM/ILM peeling as positively as expected [6, 22–24]. Differences in contractive properties of epiretinal cell proliferation might partly explain these postoperative findings and should be taken into consideration when recommending surgical intervention in eyes with LMH. In this study, half of all eyes with LHEP were accompanied by eccentric foci of conventional ERM. In these cases of LMH, macular surgery might be indicated and surgical outcome of these eyes should be addressed in further studies.

## Figures and Tables

**Table 1:** Analysis of spectral-domain optical coherence tomography (SD-OCT) and immunocytochemistry of lamellar hole-associated proliferation (LHEP) removed from eyes with lamellar macular holes (LMH).

ID number	SD-OCT analysis						Immunocytochemistry					
	LHEP	ERM	Preop defect of ellipsoid zone	Preop defect of ELM	Postop defect of ellipsoid zone	Postop defect of ELM	Anti-GFAP	Anti-CD45	Anti-CD64	Anti-*α*-SMA	Anticollagen type I	Anticollagen type II
1	+	+	+	−	+	−	+	+	+	(+)	(+)	+
2	+	+	+	+	+	−	+	+	+	+	+	+
3	+	−	+	−	+	−	++	+	+	(+)	(+)	+
4	+	+	−	−	−	−	+	+	+	+	(+)	+
5	+	−	+	−	−	−	+	(+)	+	−	+	(+)
6	+	−	+	−	+	−	++	+	(+)	(+)	+	+
7	+	+	+	+	+	+	+	+	+	−	+	+
8	+	−	+	−	+	−	++	+	+	−	+	(+)
9	+	−	+	−	+	−	+	+	(+)	−	+	+
10	+	+	−	−	−	−	++	+	+	(+)	+	+

ERM: epiretinal membrane; extrafoveal location with contractive properties; ELM: external limiting membrane; GFAP: glial fibrillary acidic protein; *α*-SMA: *α*-smooth muscle actin.
